# Supplementary material for: QRS detection in single-lead, telehealth electrocardiogram signals: Benchmarking open-source algorithms
Source: PLOS Digit Health. 2024 Aug 13;3(8):e0000538. doi: 10.1371/journal.pdig.0000538 (PMC7617317; doi:10.1371/journal.pdig.0000538)
Supplement: S1 Text — The Supplementary Material provides additional results, details of the study methodology, and links to algorithms and datasets. (PDF) [file pdig.0000538.s001.pdf]

QRS detection in single-lead, telehealth electrocardiogram signals: benchmarking open-source algorithms

Supplementary Material

Algorithm sources

Table A provides source links for the QRS detection algorithms used in this study.

Excluded algorithms

Table B summarises the QRS detectors which were excluded from this study:

- **aristot**: Excluded because only a C implementation was found, and the algorithm did not perform competitively compared to gqrs in (1).
- **match**: Excluded because the available implementation only accepted signals with a sampling frequency of either 250 Hz or 360 Hz.
- **rodr**: Excluded because the available implementation was found to predominantly lead to errors, as reported in (2). Additionally, the results in the cases where no error occurred were not competitive.
- **rsslope**: Excluded because the available implementation was found to predominantly lead to errors.
- **visgraph**: Excluded due to a substantially longer execution time than other algorithms, which was deemed undesirable for the mobile setting.
- **zong**: Excluded because the available implementation consistently led to errors during evaluation.

Dataset sources

Table C provides links to the publicly available datasets used in this study. Note that the SAFER dataset is private, and further information on this dataset is available in (3).

Threshold selection

Figure A shows the performance of QRS detectors for thresholds ranging from ± 40ms to ± 150ms. Figure A(a) and (b) show results for the TELE and SAFER datasets respectively. These were used to inform the choice of threshold to determine whether QRS complexes were correctly detected.

The positive predictive value and sensitivity of QRS detectors

Additional results for the positive predictive value (PPV) and sensitivity (SEN) of QRS detectors are provided in Figures B and C.

Comparison between males and females

Additional results comparing performance between males and females in supervised ECGs are provided in Figure D.

p-values for statistical tests

The p-values for comparisons between datasets are provided in Table D. The p-values for comparisons between male and female subjects are provided in Table E

References

[1] M. Llamedo and J. P. Martínez, "QRS detectors performance comparison in public databases," in *Computing in Cardiology*. IEEE, 2014, pp. 357–360.

[2] D. Makowski *et al.*, "NeuroKit2: A python toolbox for neurophysiological signal processing," *Behavior Research Methods*, vol. 53, no. 4, pp. 1689–1696, 2021.

[3] M. Pandiaraja *et al.*, "Screening for atrial fibrillation: Improving efficiency of manual review of handheld electrocardiograms," *Engineering Proceedings*, vol. 2, no. 1, p. 78, 2020.

[4] G. B. Moody and R. G. Mark, "Development and evaluation of a 2-lead ECG analysis program," *Computers in cardiology*, vol. 9, pp. 39–44, 1982.

[5] A. L. Goldberger *et al.*, "PhysioBank, PhysioToolkit, and PhysioNet: components of a new research resource for complex physiologic signals," *Circulation*, vol. 101, no. 23, pp. E215–220, 2000.

[6] J. Pan and W. J. Tompkins, "A real-time QRS detection algorithm," *IEEE Transactions on Biomedical Engineering*, vol. BME-32, no. 3, pp. 230–236, 1985.

[7] B. Porr and L. Howell, "py-ecg-detectors: Seven ECG heartbeat detection algorithms and heartrate variability analysis," 2022, retrieved on: 2022-08-22. [Online]. Available: https://github.com/berndporr/py-ecg-qrs-detectors

[8] T. Rodrigues *et al.*, "A low-complexity r-peak detection algorithm with adaptive thresholding for wearable devices," in *25th International Conference on Pattern Recognition*, 2021, pp. 1–8.

[9] R. Gutierrez-Rivas *et al.*, "Novel real-time low-complexity QRS complex detector based on adaptive thresholding," *IEEE Sensors Journal*, vol. 15, no. 10, pp. 6036–6043, 2015.

[10] P. Podziemski and J. Gieraltowski, "Fetal heart rate discovery: Algorithm for detection of fetal heart rate from noisy, noninvasive fetal ECG recordings," in *Computing in Cardiology*, 2013, pp. 333–336.

[11] T. Koka and M. Muma, "Fast and Sample Accurate R-Peak Detection for Noisy ECG Using Visibility Graphs," in *44th Annual International Conference of the IEEE Engineering in Medicine & Biology Society*, 2022, pp. 121–126.

[12] W. Zong *et al.*, "An open-source algorithm to detect onset of arterial blood pressure pulses," in *Computers in Cardiology, 2003*, 2003, pp. 259–262.

Table A
Source links for the QRS detection algorithms.

Shortname	Full Source Link
aristot	https://archive.physionet.org/physiotools/activity/cic92/node9.html
christ	https://github.com/berndporr/py-ecg-detectors
engz	https://github.com/berndporr/py-ecg-detectors/blob/master/ecgdetectors.py
fnvg	https://github.com/JonasEmrich/vg-beat-detectors
fwhvg	https://github.com/JonasEmrich/vg-beat-detectors
gamb	https://github.com/PIA-Group/BioSPPy/blob/master/biosppy/signals/ecg.py#L923
gqrs	https://www.physionet.org/content/wfdb-python/3.3.0/#files-panel
hamilt	https://github.com/berndporr/py-ecg-detectors
jqrs	https://github.com/alistairewj/peak-detector/blob/master/sources/qrs_detect2.m
kali	https://github.com/neuropsychology/NeuroKit/blob/master/neurokit2/ecg/ecg_peaks.py
mart	https://github.com/neuropsychology/NeuroKit/blob/master/neurokit2/ecg/ecg_peaks.py
match	https://github.com/berndporr/py-ecg-detectors/blob/master/ecgdetectors.py
nab	https://github.com/neuropsychology/NeuroKit/blob/master/neurokit2/ecg/ecg_peaks.py
nk	https://github.com/neuropsychology/NeuroKit/blob/master/neurokit2/ecg/ecg_peaks.py
pan-tomp	https://github.com/neuropsychology/NeuroKit/blob/master/neurokit2/ecg/ecg_peaks.py
rdeco	https://physionet.org/content/r-deco/1.0.0/R_peak_detection/Algorithm/peak_detection.m
rodr	https://github.com/neuropsychology/NeuroKit/blob/master/neurokit2/ecg/ecg_peaks.py
rpeak	http://www.mit.edu/~gari/CODE/ECGtools/ecgBag/rpeakdetect.m
rsslope	https://archive.physionet.org/challenge/2013/sources/
two-avg	https://github.com/berndporr/py-ecg-detectors/blob/master/ecgdetectors.py
unsw	https://dataverse.harvard.edu/dataset.xhtml?persistentId=doi:10.7910/DVN/QTG0EP
visgraph	https://github.com/taulokoka/visgraphdetector/blob/main/visgraphdetector.py
wqrs	https://github.com/berndporr/py-ecg-detectors/blob/master/ecgdetectors.py
zong	https://github.com/PIA-Group/BioSPPy/blob/master/biosppy/signals/ecg.py#L513

Table B
Excluded QRS detection algorithms

Abbreviation	Name	Reference(s)
aristot	aristotle	(4; 5)
match	matched filter	(6; 7)
rodr	rodrigues	(8; 9; 2)
rsslope	rs-slope	(10)
visgraph	visgraphdetector	(11)
zong	zong, Slope Sum Function (ssf)	(12; 2)

Table C
Links to the publicly available datasets used in this study.

Database	Full Source Link
ARR	https://www.physionet.org/physiobank/database/mitdb/
HIGH	https://physionet.org/content/challenge-2014/1.0.0/
LOW	https://physionet.org/content/challenge-2014/1.0.0/
SIN	https://physionet.org/physiobank/database/nsrdb/
TELE	https://doi.org/10.7910/DVN/QTG0EP

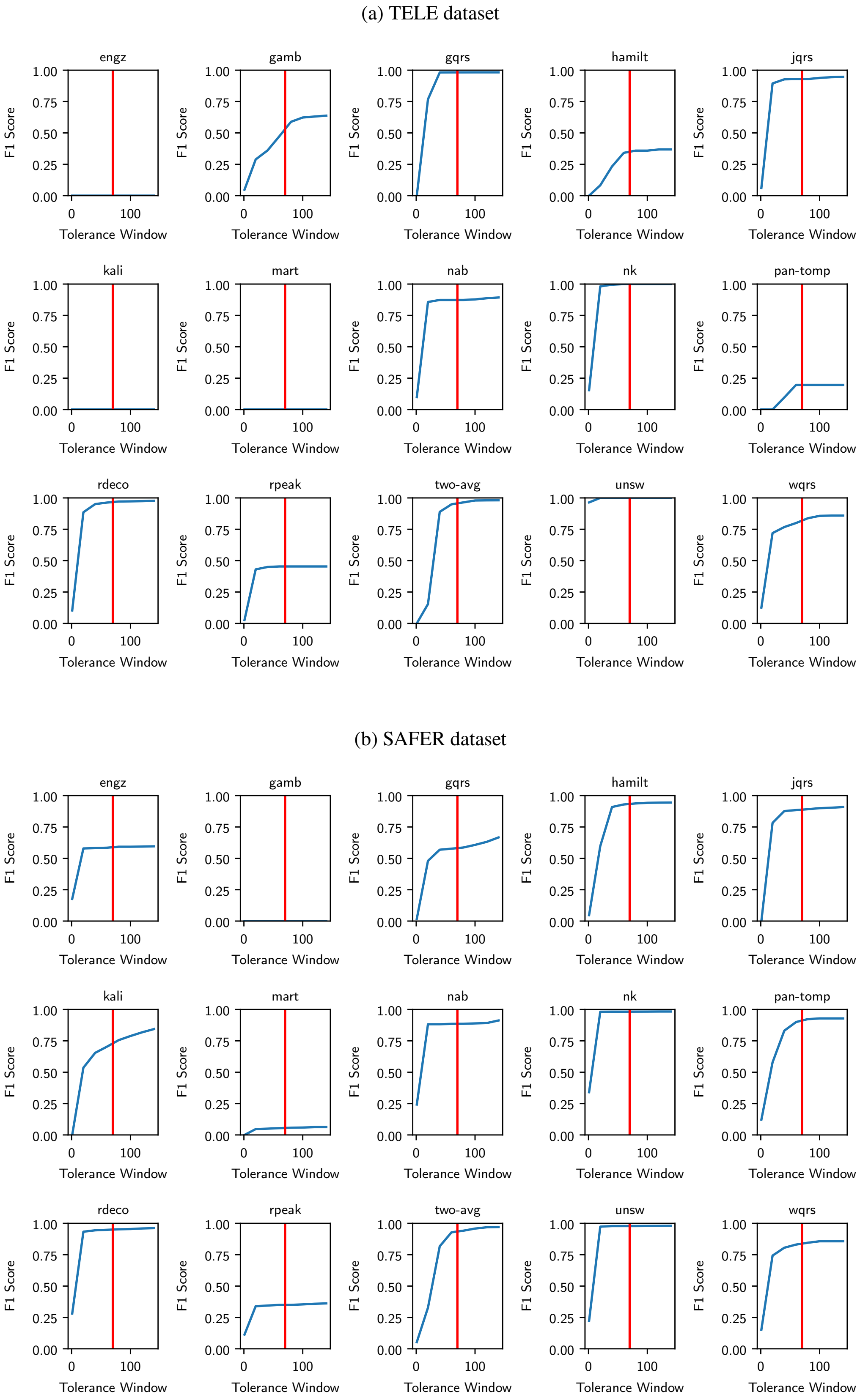

Figure A: The performance of QRS detectors when using different thresholds to identify correct QRS detections. Each plot shows the performance of a QRS detector when assessed using thresholds varying from 1 to 140ms.

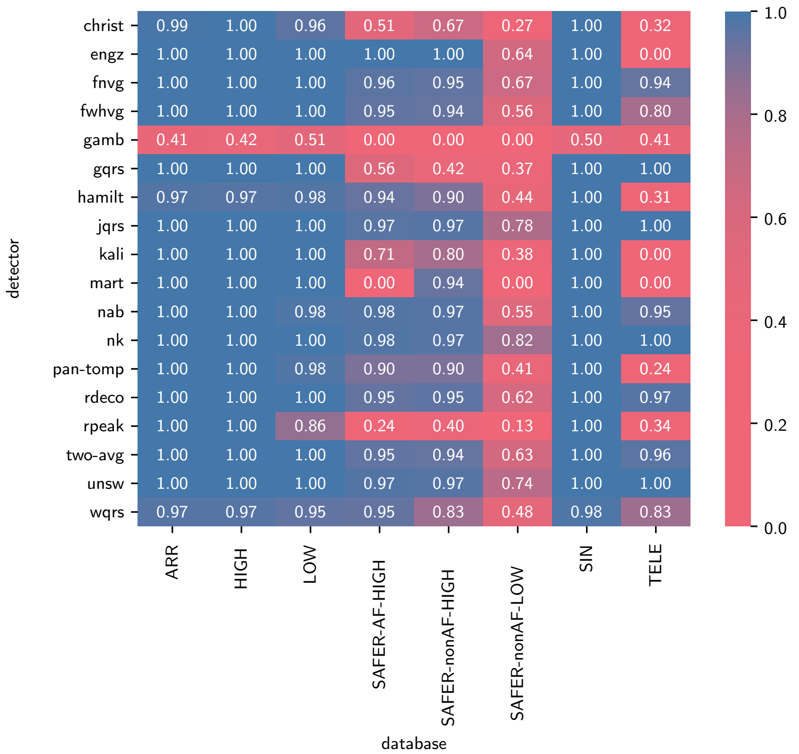

Figure B: The positive predictive value (PPV) of QRS detectors.

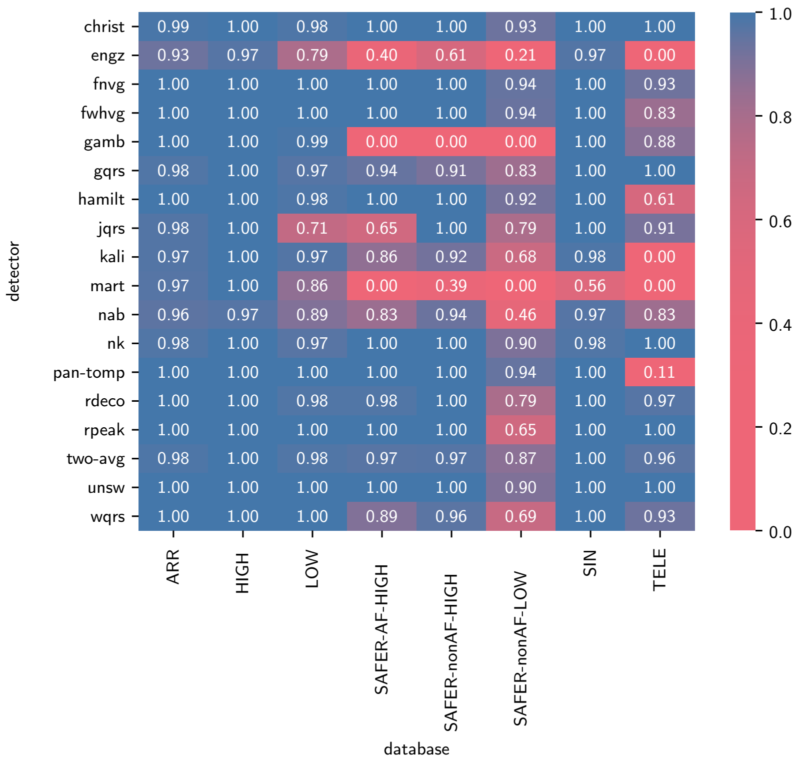

Figure C: The sensitivity (SEN) of QRS detectors.

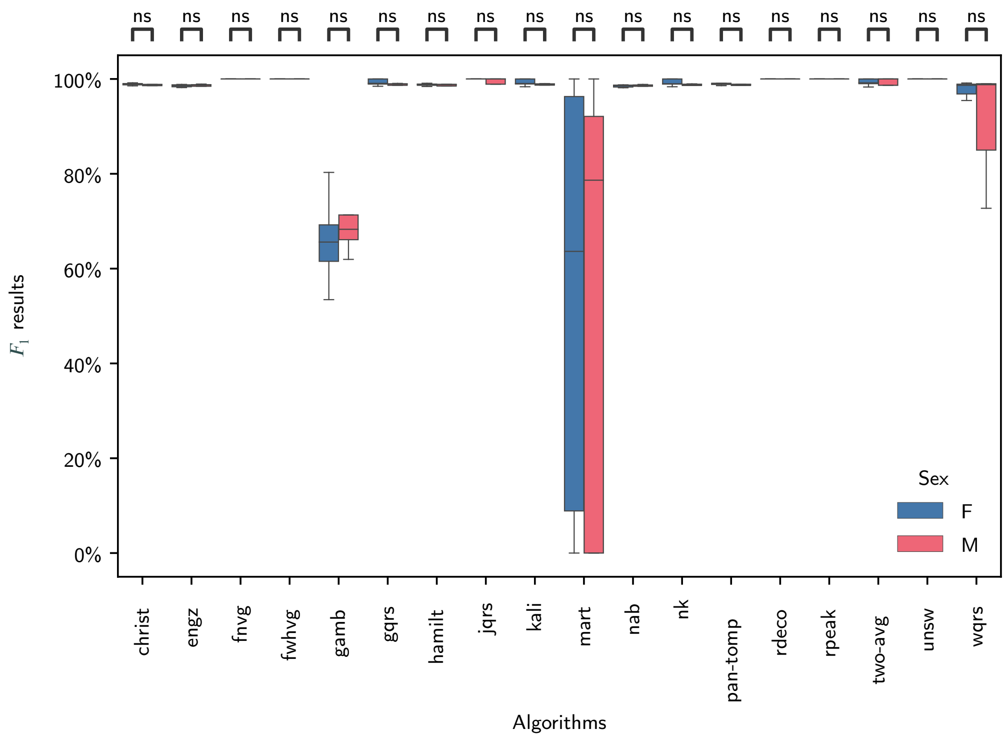

(a) SIN database (including 13 female subjects and 5 male subjects).

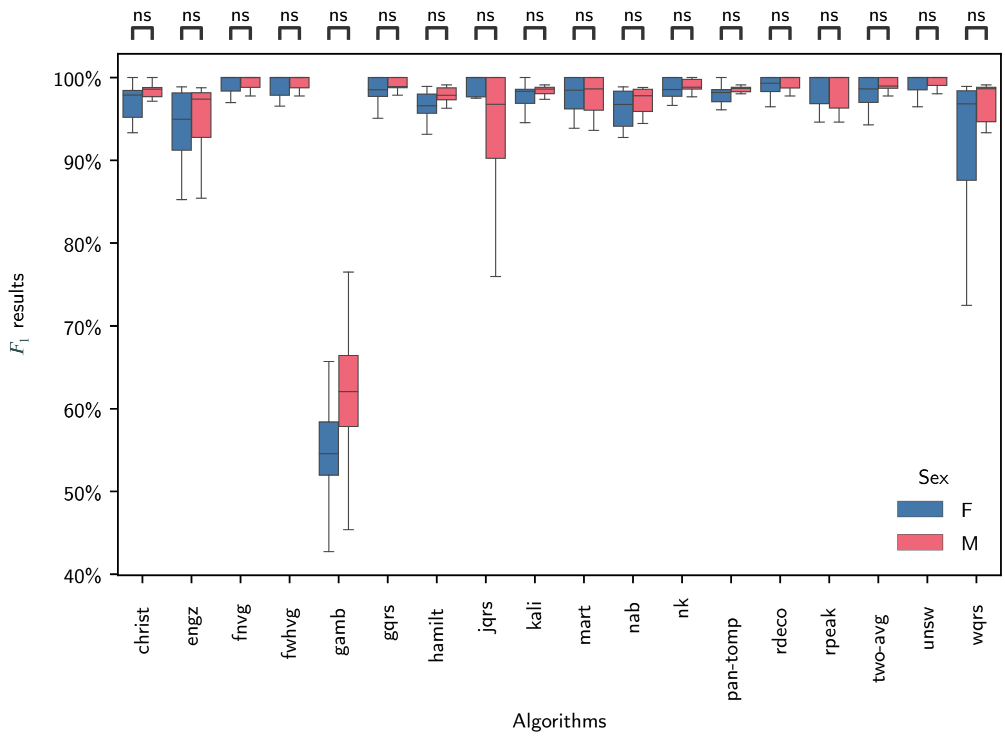

(b) ARR database (including 22 female and 26 male subjects).

Figure D: Comparison of the performance of QRS detectors between female (F) and male (M) subjects on supervised ECGs. *Definitions: * - significant difference; ns - no significant difference.*

Table D
p-values for comparisons between datasets. ** indicates a significant p-value after applying a Bonferoni correction for multiple comparisons.*

QRS Detector	Comparison					
	**ARR** vs. **SIN**	**SAFER-AF-HIGH** vs. **SAFER-nonAF-HIGH**	**SIN** vs. **SAFER-nonAF-HIGH**	**ARR** vs. **SAFER-AF-HIGH**	**SAFER-nonAF-HIGH** vs. **SAFER-nonAF-LOW**	**HIGH** vs. **LOW**
**christ**	0.004	0.218	0.000*	0.000*	0.000*	0.000*
**engz**	0.000*	0.000*	0.000*	0.000*	0.000*	0.000*
**fnvg**	0.027	0.157	0.000*	0.000*	0.000*	0.000*
**fwhvg**	0.070	0.276	0.000*	0.000*	0.000*	0.000*
**gamb**	0.001*	0.299	0.000*	0.000*	1.000	0.000*
**gqrs**	0.513	0.000*	0.000*	0.000*	0.000*	0.000*
**hamilt**	0.001*	0.002*	0.000*	0.016	0.000*	0.021
**jqrs**	0.058	0.000*	0.000*	0.000*	0.000*	0.000*
**kali**	0.000*	0.010	0.000*	0.000*	0.000*	0.000*
**mart**	0.000*	0.000*	0.535	0.000*	0.000*	0.000*
**nab**	0.004	0.000*	0.000*	0.000*	0.000*	0.000*
**nk**	0.474	0.000*	0.003*	0.397	0.000*	0.000*
**pan-tomp**	0.002*	0.232	0.000*	0.000*	0.000*	0.000*
**rdeco**	0.110	0.001*	0.000*	0.000*	0.000*	0.000*
**rpeak**	0.041	0.018	0.000*	0.000*	0.000*	0.000*
**two-avg**	0.140	0.344	0.000*	0.000*	0.000*	0.000*
**unsw**	0.300	0.055	0.000*	0.000*	0.000*	0.000*
**wqrs**	0.162	0.015	0.000*	0.000*	0.000*	0.202

Table E
p-values for comparisons between males and females. ** indicates a significant p-value after applying a Bonferoni correction for multiple comparisons.*

QRS Detector	Dataset			
	**SIN**	**ARR**	**SAFER-nonAF-HIGH**	**SAFER-AF-HIGH**
**christ**	1.000	1.000	1.000	1.000
**engz**	1.000	1.000	1.000	1.000
**fnvg**	1.000	1.000	1.000	1.000
**fwhvg**	1.000	1.000	1.000	1.000
**gamb**	1.000	0.180	1.000	1.000
**gqrs**	1.000	1.000	1.000	0.000*
**hamilt**	1.000	0.457	1.000	1.000
**jqrs**	1.000	1.000	1.000	0.000*
**kali**	1.000	1.000	1.000	1.000
**mart**	1.000	1.000	1.000	1.000
**nab**	1.000	1.000	1.000	0.534
**nk**	0.671	1.000	1.000	1.000
**pan-tomp**	1.000	0.173	1.000	1.000
**rdeco**	1.000	1.000	1.000	1.000
**rpeak**	1.000	1.000	1.000	1.000
**two-avg**	1.000	1.000	1.000	1.000
**unsw**	1.000	1.000	1.000	0.317
**wqrs**	1.000	0.610	1.000	1.000
