# Supplementary material for: QRS detection in single-lead, telehealth electrocardiogram signals: Benchmarking open-source algorithms
Source: PLOS Digit Health. 2024 Aug 13;3(8):e0000538. doi: 10.1371/journal.pdig.0000538 (PMC7617317; doi:10.1371/journal.pdig.0000538)
Supplement: Attachment — Submitted filename: Response to reviewers - 2024-04 (20240503).pdf [file pdig.0000538.s002.pdf]

**QRS detection in single-lead, telehealth electrocardiogram signals: benchmarking open-source algorithms**

**Response to Reviewer Comments**

**PDIG-D-24-00016**

We thank the reviewers for their detailed comments, which have greatly helped improve the paper. Below, we provide a point-by-point response to the comments.

## Reviewer #1:

This study undertakes an extensive statistical analysis, amalgamating QRS detectors with diverse datasets and employing F1 score and error rate as performance metrics. However, a meticulous examination of the congruence between reported results in Figure 3 and Figure 4 is recommended to ensure coherence. Specifically, the instance where both the F1 score and error rate for the cell (mart, SAFER-nonAF-LOW) are recorded as 0 necessitates careful scrutiny, as their concurrent occurrence may signal potential calculation errors.

***Response:*** *Thank you for raising this. We have now reviewed the results in Figures 3 and 4, and are confident that they are as intended. To explain, in the case of 'mart' on the SAFER-nonAF-LOW dataset, this QRS detector did not correctly identify any QRS complexes (as shown by a sensitivity of 0.00 in Figure 9). However, it did successfully execute on all of the ECGs, as indicated by an error rate of 0.0. We have now amended the caption of Figure 4 to make it clear what is meant by 'error rates' to avoid any confusion between the intended meaning (algorithms failing to execute) and the concept of errors in QRS detection. The new caption now reads as follows:*

*"The error rates for each QRS detector (expressed as percentages). These indicate the proportion of ECG windows for which the QRS detector algorithms failed to execute (i.e. encountered an error)."*

*We have also stated this in the text of Section 3.1.*

Furthermore, the application of the Mann-Whitney U test to assess performance across various subsets is acknowledged. Nonetheless, the absence of a comprehensive presentation of detailed testing results and statistical values introduces a notable limitation. To enhance the study's robustness, it is advisable to furnish explicit p-values and statistics derived from the Mann-Whitney U test. Relying solely on boxplot representations poses a potential constraint, particularly when disparities between median values seemingly contradict the non-significant (ns) result, assumed to arise from the U test. A more exhaustive reporting of statistical outcomes is imperative for a nuanced and compelling interpretation of the comparative analyses.

***Response:*** *We have now provided the p-values for all the Mann-Whitney U-tests in Tables 6 and 7 in Appendix A.7. These are referred to at the end of the Results section.*

## Reviewer #2:

This paper benchmarked 18 open-source QRS detection algorithms to identify the best-performing one for ECG signals. The study compares their performance across datasets, including a novel dataset collected during AF screening. The manuscript has good quality and the results are interesting. I have a few comments:

1. Figure 7,8,9, without any explanation. What is the purpose of doing these experiments, what message the results convey?

***Response:*** *Figures 7, 8 and 9 in the original manuscript are now figures 8, 9 and 10 in the revised manuscript (as we inserted a new figure before these). These figures all provide additional results from the experiment which were of some interest but not critical to the main conclusion. Consequently, they are provided in Appendix A.5 rather than the main text. Explanations for these three figures are provided as follows:*

- *Figures 8 and 9 are now referred to in Section 3.1 as follows: "Additional results for the positive predictive value (PPV) and sensitivity (SEN) are provided in Figures 8 and 9 in Appendix A.5. These metrics show that: gamb performed poorly because of a low PPV, indicating that it falsely detected additional QRS complexes; and mart, and engz had a low SEN, indicating that they frequently missed QRS complexes."*
- *Figure 10 is now referred to in Section 3.4 as follows: "There were no significant differences in performance between sexes on the SIN and ARR datasets (see Figures 10a and 10b in Appendix A.6), although we note the small numbers of subjects in each group in the SIN dataset (13 female and 5 male)."*

2. What is the computing environment of running python and matlab program? How many CPU cores are used? Are there any parallelization used?

***Response:*** *We have now provided details of the computing environment in Section 2.4 as follows:*

*"Execution times were assessed as the median time taken for an algorithm to process each 30s ECG segment. The analysis was performed on a MacBook Air (M1, 2020, 16 GB RAM, 8 cores) without parallelization. The assessment was run in Visual Studio Code 1.73.0, using Python 3.9, and calling MATLAB R2022a for QRS detectors written in MATLAB code."*

3. Could the authors specify what are the number of male/female samples in each dataset?

***Response:*** *We have now elaborated on this in Section 2.4. Briefly, in the descriptions of the datasets we provide the number of male and female samples for the SIN, ARR and SAFER datasets, but not for the HIGH, LOW and TELE datasets. We have checked, and to hte best of our knowledge no information on gender is provided in HIGH, LOW, or TELE. Therefore, we have clarified this in Section 2.4, stating:*

*"Comparisons between female and male subjects were made on the SIN, ARR and SAFER datasets, but not on the HIGH, LOW and TELE datasets as to the best of our knowledge they do not contain information on gender."*

## Reviewer #3:

This work describes the performance of several QRS detection algorithms. While the work is not novel, in and of itself, there certainly is a welcome place in the literature for a thorough evaluation of these approaches. As the authors' note, QRS detection forms an important part of several clinically relevant tasks (e.g., arrhythmia detection, HRV analyses, etc.). However, the work suffers from technical flaws that require clarification before it can be published.

1) The authors definitions of sensitivity and specificity are not correct as written and it is not clear whether this represents a typographical error or a true error in how these values were calculated. For example, the authors suggest that the numerator for both sensitivity calculations and for positive predictive value calculations are the same. This is incorrect. The sensitivity is the true positive rate and therefore the numerator refers to the number of reference QRS annotations that are also correct according to the algorithm. By contrast, numerator in the equation for the positive predictive value (PPV) is the number of algorithmic predictions that are correct. This is a very important point that needs to be clarified/corrected.

***Response:*** *We have reviewed the definitions provided for sensitivity and specificity in Section 2.4 (equations 1 and 2), and acknowledge that these were not entirely clear. Therefore, we have elaborated on the definitions, by now providing the definitions in both the usual nomenclature (e.g. true positives, TP, and false positives, FP), and our amended nomenclature which is designed to make it easier for the reader to understand the statistics in the context of QRS detection. We have also clarified in the text immediately above the equations the analogies between the usual nomenclature and our amended nomenclature. The text now reads as follows:*

*"the number of reference QRS complex annotations ($nref$ , corresponding to the number of actual positives, P); the number of QRS complexes identified by an algorithm ($nalg$ , corresponding to the number of predicted positives, i.e. true positives + false positives, TP+FP); and the number of QRS complexes which were correctly identified ($ncorrect$, corresponding to the number of true positives, TP)"*

$$SEN(\%) = \frac{TP}{P} \times 100 = \frac{n_{correct}}{n_{ref}} \times 100 \quad (1)$$

$$PPV(\%) = \frac{TP}{TP + FP} \times 100 = \frac{n_{correct}}{n_{alg}} \times 100 \quad (2)$$

$$F_1(\%) = \frac{2 \times PPV \times SEN}{PPV + SEN} \times 100 \quad (3)$$

*As regards the reviewer's comment that "the authors suggest that the numerator for both sensitivity calculations and for positive predictive value calculations are the same. This is incorrect." We respectfully disagree with this statement. For instance, in their article titled 'Foundational Statistical Principles in Medical Research: Sensitivity, Specificity, Positive Predictive Value, and Negative Predictive Value' ( https://doi.org/10.3390/medicina57050503 ), Monaghan et al. state the equations for sensitivity and positive predictive value as:*

- *"Sensitivity =TP/(TP + FN)"*
- *"PPV =TP/(TP + FP)"*

*This indicates that these statistics can be expressed with the same numerator. If we have misunderstood the reviewer's comment then we'll be happy to discuss further.*

2) The authors define a correct prediction as one that is within +/- 150ms of the reference QRS annotation. This is a large range. In the introduction, the authors point to HRV and arrhythmia detection as important tasks that depend on QRS detection. However, such a large range (+/- 150ms of the QRS reference) will certainly not lead to accurate HRV estimates and will certainly not help with arrhythmia detection. The fact that this has been used in other studies is a poor reason to reply on this standard here. It is imperative that the authors discuss results using cutoffs that will yield more reliable HRV estimates (e.g., +/- 40ms).

***Response:*** *Thank you for this very helpful comment. We have now investigated different potential thresholds for defining a correct prediction. Based on this analysis, alongside logical reasoning, we have now changed the threshold from +/- 150ms to +/- 75ms, and have updated the results accordingly. This resulted in a minor modification of the conclusion, from the previous conclusion that "The Neurokit, 'two average', and University of New South Wales QRS detectors performed best in this study" to "The Neurokit and University of New South Wales QRS detectors performed best in this study" [as stated in the abstract]. We have now explained the reasons for changing the threshold from +/- 150ms to +/- 75ms in the manuscript, as follows:*

*“A threshold of ± 75ms was chosen to classify QRS detections as correct or not for the following reasons. QRS complexes typically last <120ms in health (36), although can last longer in disease (37). ± 75ms was identified as a conservative threshold which would classify any QRS detections lying on a QRS complex as correct, whilst classifying any detections on other ECG waves (such as p- or t-waves) as incorrect. This was based on the assumptions that: a QRS complex lasts up to approximately 150ms; the R-wave is located approximately in the centre of a QRS complex; and reference QRS annotations are at the locations of Rwaves. To investigate the suitability of this threshold, we assessed the performance of the QRS detectors on the telehealth (TELE and SAFER) datasets for thresholds ranging from ± 1 to 140ms. The results (shown in Appendix A.4) show that for all QRS detectors performance was poorer at low thresholds, with performance generally approaching a maximum between 20 and 100ms (such as ≈ 20ms for nk and unsw, and ≈ 60-80ms for pan-tomp and two-avg). Therefore, a threshold of ± 75ms appeared a reasonable choice. In comparison, previous work in this area has used tolerances of 50 ms (5) and ± 150 ms (19).”*

*Thank you too for the comment on heart rate variability analysis and arrhythmia detection. Since the current manuscript focuses on QRS detection rather than its application, we have now discussed these aspects as limitations in Section 4.3:*

*“Furthermore, we did not investigate the accuracy of RR-intervals derived from QRS detections, nor their suitability for heart rate variability analysis or arrhythmia detection. We note that additional processing steps may be required to accurately derive RR-intervals, such as locating the R-wave on each detected QRS complex.”*
